# TRANSFORMING TELEHEALTH ACCESS: INTEGRATING BEHAVIORAL HEALTH AND TECHNOLOGY COACHING

**DOI:** 10.1093/geroni/igae098.3442

**Published:** 2024-12-31

**Authors:** Pedro Carbajal-Madrid, Rachel Holben

**Affiliations:** SCAN Health Plan, Long Beach, California, United States; SCAN Health Plan, Long Beach, California, United States

## Abstract

Research indicates only 21% of older adults in the U.S. utilize telehealth services, with economic, linguistic or literacy challenges exacerbating barriers to accessing quality health care. The Insights Program at SCAN / Independence at Home is a behavioral health program that provides individual and group therapy for people aged 55 and over and their caregivers. Serving over 2,400 individuals in 2023, the Insights program offers no cost services to those who otherwise may not be able to access mental health treatment, with a reduction in symtoms of depression and anxiety over 50%. During the Covid-19 pandemic, transitioning from in-person to telehealth sessions highlighted clients’ struggles with technology as they were expected to engage with online platforms without prior training. This led to older adults avoiding scheduling appointments or missing scheduled visits due to a lack of understanding of technology. To meet this unmet need, Independence at Home launched the T.E.C (Technology, Education and Coaching) program in 2021. This program pairs trained Digital Coaches with Insights’ clients, providing individualized support to help them use virtual platforms for online therapy sessions and access other health and social services. Early outcomes show the T.E.C program significantly increased digital literacy (1.13 vs 2.94, p<.0001 [eHealth Literacy Scale]) and technology engagement (.44 vs.75, p=.0014 [Program’s Technology Engagement Survey]) based on pre (n=22) and post (n=22) testing. These findings demonstrate the program’s positive impact on the clients’ understanding and proficiency in utilizing technology and navigating electronic health platforms.

